# CT guided cryoablation for locally recurrent or metastatic bone and soft tissue tumor: initial experience

**DOI:** 10.1186/s12885-016-2852-6

**Published:** 2016-10-13

**Authors:** Michiro Susa, Kazutaka Kikuta, Robert Nakayama, Kazumasa Nishimoto, Keisuke Horiuchi, Sota Oguro, Masanori Inoue, Hideki Yashiro, Seishi Nakatsuka, Masaya Nakamura, Morio Matsumoto, Kazuhiro Chiba, Hideo Morioka

**Affiliations:** 1 Department of Orthopaedic Surgery, Keio University School of Medicine, Shinjuku-ku, Tokyo, 160-8582 Japan; 2 Department of Orthopaedic Surgery, National Defense Medical College, Tokorozawa, Saitama 359-8513 Japan; 3 Department of Diagnostic Radiology, School of Medicine, Keio University School of Medicine, Shinjuku-ku, Tokyo, 160-8582 Japan

**Keywords:** Cryoablation, Bone tumor, Soft tissue tumor, Metastasis

## Abstract

**Background:**

Historically, local control of recurrent sarcomas has been limited to radiotherapy when surgical re-resection is not feasible. For metastatic carcinomas to the bone or soft tissue, radiotherapy and some interventional radiology treatment along with other systemic therapies have been widely advocated due to the possibility of disseminated disease. These techniques are effective in alleviating pain and achieving local control for some tumor types, but it has not been effective for prolonged local control of most tumors. Recently, cryoablation has been reported to have satisfactory results in lung and liver carcinoma treatment. In this study, we analyzed the clinical outcome of CT-guided cryoablation for malignant bone and soft tissue tumors to elucidate potential problems associated with this procedure.

**Methods:**

Since 2011, 11 CT-guided cryoablations in 9 patients were performed for locally recurrent or metastatic bone and soft tissue tumors (7 males and 2 females) at our institute. The patients’ average age was 74.8 years (range 61–86) and the median follow up period was 24.1 months (range 5–48). Histological diagnosis included renal cell carcinoma (*n* = 4), dedifferentiated liposarcoma (*n* = 2), myxofibrosarcoma (*n* = 2), chordoma (*n* = 1), hepatocellular carcinoma (*n* = 1), and thyroid carcinoma (*n* = 1). Cryoablation methods, clinical outcomes, complications, and oncological outcomes were analyzed.

**Results:**

There were 5 recurrent tumors and 6 metastatic tumors, and all cases had contraindication to either surgery, chemotherapy or radiotherapy. Two and 3 cycles of cryoablation were performed for bone and soft tissue tumors, respectively. The average length of the procedure was 101.1 min (range 63–187), and the average number of probes was 2.4 (range 2–3). Complications included 1 case of urinary retention in a patient with sacral chordoma who underwent prior carbon ion radiotherapy, 1 transient femoral nerve palsy, and 1 minor wound complication. At the final follow up, 4 patients showed no evidence of disease, 2 were alive with disease, and 3 died of disease.

**Conclusions:**

Reports regarding CT-guided cryoablation for musculoskeletal tumors are rare and the clinical outcomes have not been extensively studied. In our case series, CT-guided cryoablation had analgesic efficacy and there were no cases of local recurrence post procedure during the follow-up period. Although collection of further data regarding use of this technique is necessary, our data suggest that cryoablation is a promising option in medically inoperable musculoskeletal tumors.

## Background

Recurrent and metastatic bone and soft tissue tumors pose significant problems because of their refractory nature. Re-resection of local sarcoma recurrence has the potential to cure the patient, but it is often difficult to treat because it is almost impossible to discern the extent of tumor infiltration after multiple operations [1]. Metastasis of carcinoma to the bone and soft tissue is also challenging due to the morbidity of patients. Various radiotherapy techniques have been reported to be effective in alleviating pain and achieving local control. Approximately 60 % of patients reportedly experience pain relief after radiotherapy [2]. Recently, carbon ion radiotherapy has been reported to be effective for local control of certain unresectable sarcomas, but its long-term outcomes require further analysis [3]. When radiation therapy is contraindicated for specific reasons, locally recurrent or metastatic bone and soft tissue tumor are often treated by palliative care.

Cryoablation is a therapeutic procedure wherein hollow cryoprobes are inserted into the tumor, causing a decrease in the local temperature below 40° C. Freezing for longer than 1 min causes cell death and subsequent thawing further ensures disruption of the cellular integrity [4, 5]. Its effectiveness for alleviating pain has been reported in musculoskeletal metastases from carcinomas, but its utility in the curative intent for metastases and local recurrence is scarce. Furthermore, only 2 case series have reported the effectiveness of cryoablation for primary musculoskeletal tumors [6, 7].

The purpose of this study was to assess the feasibility, safety and efficacy of cryosurgical ablation for locally recurrent sarcoma and metastatic carcinomas.

## Methods

**Fig. 1:**
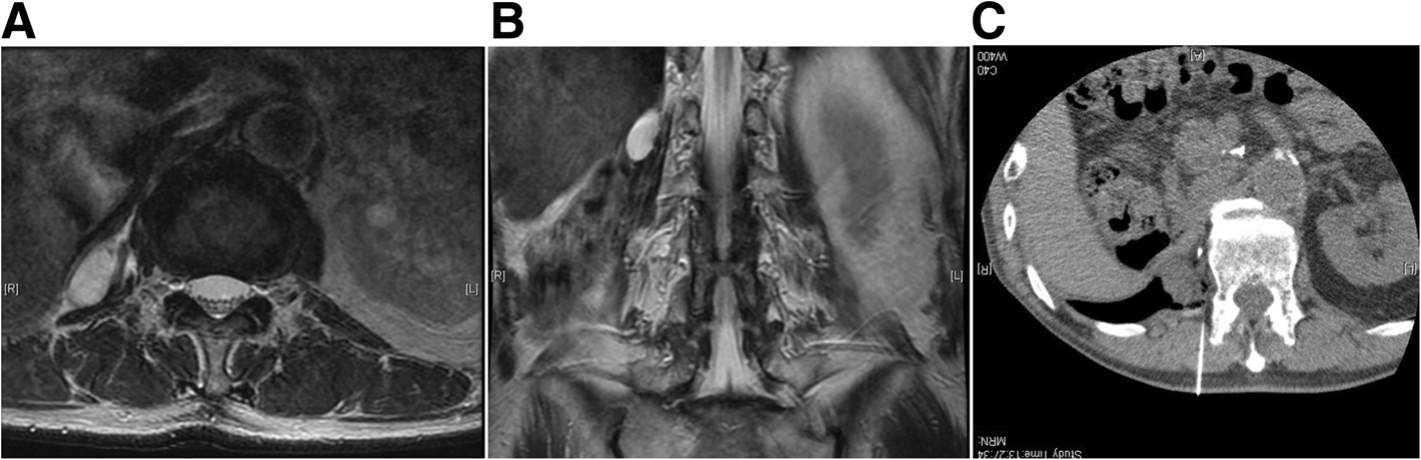
Locally recurrent, 3 cm, dedifferentiated liposarcoma in the retroperitoneal paravertebral region 1 year after initial surgery (**a**, **b**). **c** Under CT guidance, air was transperitoneally infused to displace the bowel just adjacent to the recurrent tumor

**Fig. 2:**
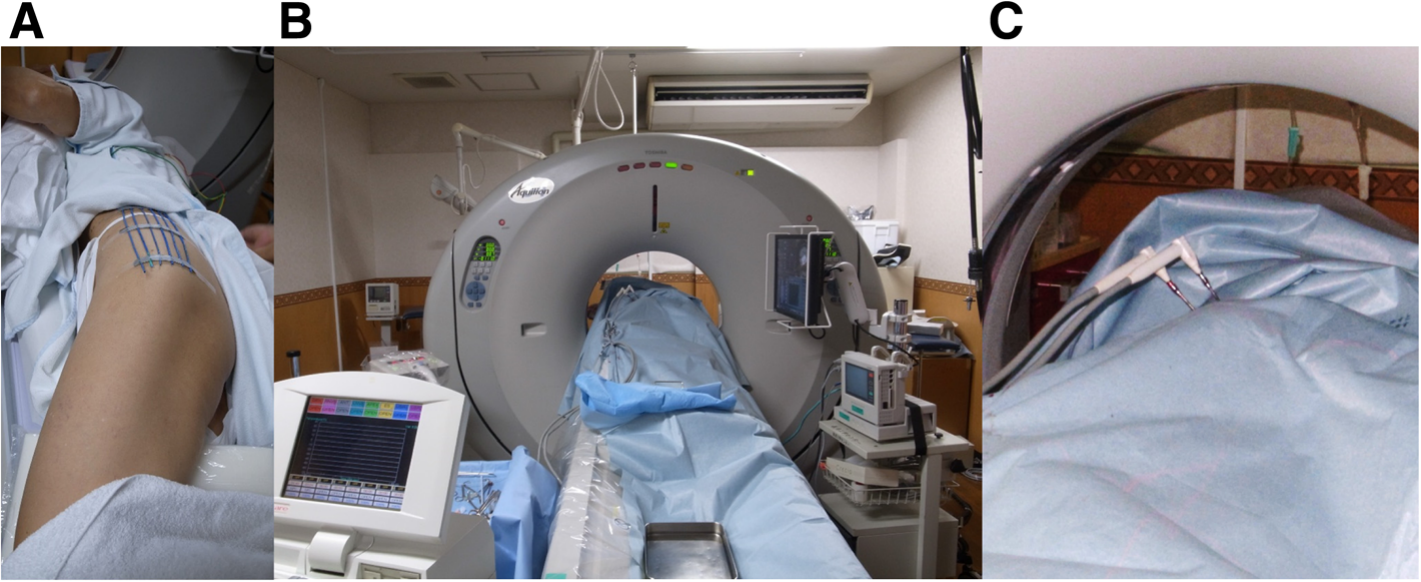
**a**, **b** Markers were placed on the left buttock to discern the optimal direction of probe insertion and cryoablation was performed under CT guidance. **c** Two probes were inserted into the lesion, and an ice ball was monitored to secure at least a 1-cm margin around the tumor

**Fig. 3:**
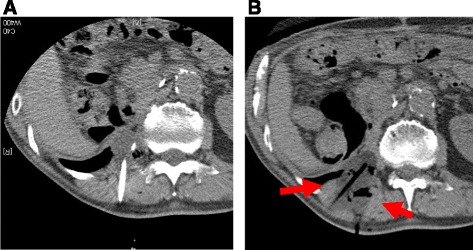
**a**, **b**. Multiple synergistic probes were inserted into the tumor to ensure complete encapsulation of the lesion by the ice ball. The ice ball was periodically monitored using CT to secure at least a 1-cm margin around the tumor

The inclusion criteria included limited symptomatic metastasis, soft tissue recurrence or recurrent skeletal disease with either osteolytic or mixed osteolytic - osteoblastic features. All the cases were contraindicated for other treatments, such as surgery or radiotherapy, due to comorbidity or prior irradiation. Specifically, the reason behind cryoablation for sarcoma cases were as follows: chordoma patient had underwent previous carbon ion radiation therapy, dedifferentiated liposarcoma of the retroperitoneal was an 86-year-old male who was determined to be inoperable due to his comorbidity, and myxofibrosarcoma patient had undergone 4 previous surgeries and refused further aggressive treatment such as an amputation. Osteoblastic lesions were excluded because local anesthesia was deemed insufficient to establish a tract for placing the ablation device. All lesions that were adjacent to vital structures such as the nerves and bowels were excluded, unless they could be displaced by injection of air to ensure safety (Fig. 1). Laboratory examination was performed to rule out any coagulation disease or infection. In total, 9 patients underwent argon - helium cryoablation for difficult tumors since 2011. The treatment was performed according to the approval of Institutional Board of Keio University School of Medicine (#20110088) because this treatment has not been validated for these tumors in Japan. Written informed consent was obtained from each participant in accordance with the Declaration of Helsinki. Cryoablation was performed on patients under local anesthesia, with an argon - helium gas based CRYO Care System (Endocare, Irvine, CA), and cryoprobes were utilized in a multiple freeze - thaw cycle that was monitored with CT guidance (Fig. 2). Multiple probes were placed to encompass the entire lesion and 2 or 3 freeze - thaw cycles (20 min freeze followed by 10 min thaw) were then performed. Each cycle reached a temperature of -196 °C at the probe tip. During cryoablation, the ice ball was periodically monitored using CT to secure at least a 1-cm margin around the tumor. The freezing duration was slightly adjusted to allow for complete tumor coverage and avoidance of adjacent critical structures (Fig. 3). One freeze - thaw cycle was added for soft tissue tumors to ensure complete tumor death in the context of the lower thermal conductivity of fat tissue that usually surrounds the tumor [8]. Upon removal of the probes, the tracts were evaluated for possible bleeding, and anticoagulant was used whenever there was bleeding from the cylinders. The average tumor size was 39.6 mm (range 22–52 mm), and tumors were localized in the ilium (*n* = 5), retroperitoneum (*n* = 2), thigh (*n* = 2), rib (*n* = 1), and sacrum (*n* = 1). Serial MRI was analyzed post cryoablation after 1, 3, 6, and 12 months. Lesions were defined as locally controlled if there was no focal enlargement according to the Response Evaluation Criteria in Solid Tumors (version 1.1) [9] and if there was no enhancement of the lesion upon gadolinium administration on MRI. The Kaplan - Meier method was used to determine the survival rate (GraphPad PRISM®4 software, GraphPad Software, San Diego, CA).

## Results

**Fig. 4:**
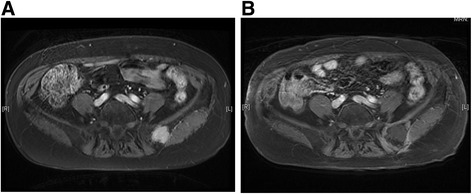
Gadolinium enhanced T1 - weighted MR image with fat suppression of an iliac metastasis from thyroid carcinoma (**a**) and 6 months after cryoablation (**b**). The effect of cryoablation is depicted as a low-signal intensity area with rim enhancement. The low-intensity area persisted during the 3 - year follow - up, a result consistent with no local recurrence

**Fig. 5:**
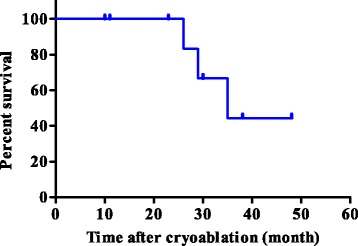
Kaplan - Meier survival curve analysis of the entire group. There was no local recurrence after cryoablation and the median survival was 35 months

During the study period, 11 lesions from 9 patients were treated using CT-guided cryoablation. Five recurrent cases were all due to sarcoma. Three cases (2 renal cell carcinoma cases and one hepatocellular carcinoma case) underwent pre - procedural transcatheter arterial embolization for preventing bleeding during cryoablation. Two and 3 cycles of cryoablation were performed for bone and soft tissue tumors, respectively. Technically, in all procedures, the ice ball successfully encompassed the entire tumor, visualized as a low density region under CT. The average procedure length was 101.1 min (range 63–187) and the average number of utilized probes was 2.4 (range 2–3). Inflammation, depicted as a high intensity area on MRI from the procedure, persisted for several months. However, at the final follow - up, only the low-intensity region that was not enhanced after gadolinium application persisted (Fig. 4). The complications included 1 case of urinary retention in a patient with sacral chordoma who underwent prior carbon ion radiotherapy, 1 transient femoral nerve palsy, and 1 minor wound complication. No post - cryoablation hemorrhage was observed, even for highly vascular lesions, which was possibly due to transcatheter arterial embolization performed prior to the procedure. All patients were discharged 1 day after the procedure. Local control was obtained in all cases; however, 2 patients (1 dedifferentiated liposarcoma and 1 myxofibrosarcoma) developed local recurrence apart from the initial procedure. Both the patients subsequently underwent a second cryoablation. At the final follow - up, 4 patients showed no evidence of disease and 2 were alive with disease, but 3 patients died due to systemic tumor metastasis. The median survival for the entire group was 35 months (Fig. 5).

## Discussion

Local control is typically achieved in approximately 60–90 % of sarcomas after wide resection, depending on the tumor location [10, 11]. Various methods, from local adjuvant therapies to novel molecular therapeutics, have the potential to further improve the outcome; however, the prognosis has not improved in the past decades possibly due to the difficulty when facing local recurrence and distant metastasis [12]. Although re-resection with or without radiotherapy is the gold standard for local recurrence of sarcomas, further surgery is often not feasible because of the uncertain spread of the lesion under imaging, the lack of an adequate barrier in additional wide resection and patient morbidity after multiple operations and systemic therapies [13, 14] . Furthermore, bone metastasis of sarcomas is a debilitating event that impairs the quality of life. Local recurrences have been reported to worsen the outcome due to consequent metastasis [15]. Metastasis of carcinomas is also difficult to treat, owing to patient morbidity after extensive treatment of the primary lesion. Carcinomas usually present with systematic metastasis which limits the treatment to radiotherapy or various interventional radiology treatment. The outcome of bone metastasis has seen great improvement through the advent of bone modifying agents such as bisphosphonates and anti - RANKL antibody, denosumab. However, there are also side effects, such as osteonecrosis of the jaw and atypical fractures. Metastasis to soft tissue is problematic when it causes a mass effect or compression of vital organs. Some cancers have improved after excision of the tumor bulk, such as in oligometastatic cases, but surgery is often challenging when it occurs in deep-seated locations. Reducing the sarcoma burden has been implicated as an adjunct to aggressive salvage chemotherapy [16, 17]. With the advent of newer drugs targeting molecular susceptibility, novel minimally invasive techniques, such as cryoablation, could be integrated for future treatment.

There has been wide variety of image guided thermal ablations, including radiofrequency, microwave, laser, high intensity ultrasound, irreversible electroporation, and cryotherapy. Percutaneous thermal ablation offers minimal invasive procedures, but each technique has their own clinical applications. Radiofrequency was the first reported percutaneous thermal ablation technique, and its efficacy has been widely reported for various tumor types. It is especially effective for small lesions, such as osteoid osteoma, for which the heat is sufficient to eradicate lesions that are usually less than 2 cm in diameter [18]. However, a limitation is that the area of ablation is not readily visualized under imaging modalities [19]. Other techniques that utilize laser and ultrasound technology have also shown efficacy, albeit in small case series for musculoskeletal tumors. Laser ablation is primarily employed in small lesions, such as osteoid osteoma, and high - intensity focused ultrasound is limited to surface lesions because an appropriate acoustic window under MRI is necessary for this technology [20].

Cryotherapy was first reported in the mid - 19th century [21], and its percutaneous application was subsequently modified for musculoskeletal tumors [22]. Cryotherapy has a strong advantage in that it can treat irregularly shaped lesions by using multiple synergistic probes as well as monitor the area of ablation, as an ice ball, to ensure accuracy. Furthermore, unlike with radiotherapy, it can be applied repeatedly. Additionally, the post procedural pain is minimal, and recovery is swift with the possible additive effect of a systemic antitumor immune response by cryoablation [23].

The effectiveness of cryoablation for palliating pain from cancer metastasis has been reported by several groups [20, 24]. Furthermore, cryoablation has been able to achieve local control of asymptomatic cancer metastasis with limited systematic spread. In a single center retrospective study, local control was achieved for over 85 % of metastases at a median time of 21 months [25]. Additionally, cryoablation for oligometastatic renal cell carcinoma has been implicated to result in higher overall survival compared to systematic treatments alone [26]. Recently, there have been sporadic reports of the use of cryoablation for soft tissue sarcomas [22, 27]. In a recent feasibility study with soft tissue sarcoma relapse, location in the wall of the trunk, shoulders and pelvic girdle; tumors with local aggressiveness; deep tumors or tumors less or equal to 5 cm, and liposarcoma and myxofibrosarcoma were deemed suitable for cryoablation [27]. Although the study populations are generally small, improved local control, analgesic efficacy, reduced complication and reduction of convalescence after the procedure has been reported [28].

Clinically, the disadvantages of cryoablation include its medical cost (it is not covered by the National Health Insurance in Japan), the necessity of an argon gas supply, equipment maintenance, and a large storage area [29]. Technically, there are several limitations to this procedure: the lesion should have an adequate distance from the skin, neurovascular structure and other viscera, and should not be localized in a weight - bearing bone. The average time of the procedure has been longer than for other percutaneous thermal ablation techniques performed at our institute (data not shown).

There are a few limitations in the present study. Most critically, the sample size is relatively small, as is the case for past reports using a small case series. The wide differences in follow - up duration stems from the lack of insurance coverage for this procedure in Japan which costs approximately 9000 U.S. dollars. This has been the major obstacle in recruiting large number of patients. In addition, the results may have been biased because only relatively small sized lesions were included in the study. Nevertheless, ablation for asymptomatic metastases or local recurrence for local control has not been thoroughly reported, and our data should provide a basis for further clinical studies to clarify the efficacy of this technique in treating such patients.

## Conclusion

The outcomes from this study suggest that cryoablation is clinically safe and feasible, because no local recurrence was observed in any of the cases. Further prospective randomized studies are warranted to elucidate the optimum protocol for this technique and effectiveness for a variety of histologically different tumors.

## References

[1] Pretell-Mazzini J, Barton MD, Conway SA, Temple HT (2015). Unplanned excision of soft-tissue sarcomas: current concepts for management and prognosis. J Bone Joint Surg Am.

[2] Wu JS, Wong R, Johnston M, Bezjak A, Whelan T (2003). Meta-analysis of dose-fractionation radiotherapy trials for the palliation of painful bone metastases. Int J Radiat Oncol Biol Phys.

[3] Matsumoto K, Imai R, Kamada T, Maruyama K, Tsuji H, Tsujii H (2013). Impact of carbon ion radiotherapy for primary spinal sarcoma. Cancer.

[4] Baust JG, Gage AA, Bjerklund Johansen TE, Baust JM (2014). Mechanisms of cryoablation: clinical consequences on malignant tumors. Cryobiology.

[5] Gage AA, Baust JM, Baust JG (2009). Experimental cryosurgery investigations in vivo. Cryobiology.

[6] Kujak JL, Liu PT, Johnson GB, Callstrom MR (2010). Early experience with percutaneous cryoablation of extra-abdominal desmoid tumors. Skeletal Radiol.

[7] Kurup AN, Woodrum DA, Morris JM, Atwell TD, Schmit GD, Welch TJ (2012). Cryoablation of recurrent sacrococcygeal tumors. J Vasc Interv Radiol.

[8] Holman WL, Kirklin JK, Anderson PG, Pacifico AD (1992). Variation in cryolesion penetration due to probe size and tissue thermal conductivity. Ann Thorac Surg.

[9] Eisenhauer EA, Therasse P, Bogaerts J, Schwartz LH, Sargent D, Ford R (2009). New response evaluation criteria in solid tumours: revised RECIST guideline (version 1.1). Eur J Cancer.

[10] Mullen JT, Hornicek FJ, Harmon DC, Raskin KA, Chen YL, Szymonifka J (2014). Prognostic significance of treatment-induced pathologic necrosis in extremity and truncal soft tissue sarcoma after neoadjuvant chemoradiotherapy. Cancer.

[11] Alldinger I, Yang Q, Pilarsky C, Saeger HD, Knoefel WT, Peiper M (2006). Retroperitoneal soft tissue sarcomas: prognosis and treatment of primary and recurrent disease in 117 patients. Anticancer Res.

[12] Mulder RL, Paulides M, Langer T, Kremer LC, van Dalen EC (2012). Cyclophosphamide versus ifosfamide for paediatric and young adult bone and soft tissue sarcoma patients. Cochrane Database Syst Rev.

[13] Emrich LJ, Ruka W, Driscoll DL, Karakousis CP (1989). The effect of local recurrence on survival time in adult high-grade soft tissue sarcomas. J Clin Epidemiol.

[14] Fiore M, Casali PG, Miceli R, Mariani L, Bertulli R, Lozza L (2006). Prognostic effect of re-excision in adult soft tissue sarcoma of the extremity. Ann Surg Oncol.

[15] Trovik CS, Gustafson P, Bauer HC, Saeter G, Klepp R, Berlin O (2000). Consequences of local recurrence of soft tissue sarcoma: 205 patients from the Scandinavian Sarcoma Group Register. Acta Orthop Scand.

[16] Yang RS, Eckardt JJ, Eilber FR, Rosen G, Forscher CA, Dorey FJ (1995). Surgical indications for Ewing's sarcoma of the pelvis. Cancer.

[17] Bacci G, Picci P, Gitelis S, Borghi A, Campanacci M (1982). The treatment of localized Ewing's sarcoma: the experience at the Istituto Ortopedico Rizzoli in 163 cases treated with and without adjuvant chemotherapy. Cancer.

[18] Lindner NJ, Ozaki T, Roedl R, Gosheger G, Winkelmann W, Wortler K (2001). Percutaneous radiofrequency ablation in osteoid osteoma. J Bone Joint Surg (Br).

[19] Rosenthal DI, Hornicek FJ, Torriani M, Gebhardt MC, Mankin HJ (2003). Osteoid osteoma: percutaneous treatment with radiofrequency energy. Radiology.

[20] Kurup AN, Callstrom MR (2010). Ablation of skeletal metastases: current status. J Vasc Interv Radiol.

[21] Gage AA (1998). History of cryosurgery. Semin Surg Oncol.

[22] Menendez LR, Tan MS, Kiyabu MT, Chawla SP (1999). Cryosurgical ablation of soft tissue sarcomas: a phase I trial of feasibility and safety. Cancer.

[23] Joosten JJ, Muijen GN, Wobbes T, Ruers TJ (2001). In vivo destruction of tumor tissue by cryoablation can induce inhibition of secondary tumor growth: an experimental study. Cryobiology.

[24] Callstrom MR, Dupuy DE, Solomon SB, Beres RA, Littrup PJ, Davis KW (2013). Percutaneous image-guided cryoablation of painful metastases involving bone: multicenter trial. Cancer.

[25] McMenomy BP, Kurup AN, Johnson GB, Carter RE, McWilliams RR, Markovic SN (2013). Percutaneous cryoablation of musculoskeletal oligometastatic disease for complete remission. J Vasc Interv Radiol.

[26] Bang HJ, Littrup PJ, Goodrich DJ, Currier BP, Aoun HD, Heilbrun LK (2012). Percutaneous cryoablation of metastatic renal cell carcinoma for local tumor control: feasibility, outcomes, and estimated cost-effectiveness for palliation. J Vasc Interv Radiol.

[27] Lippa N, Sargos P, Italiano A, Kind M, Dallaudiere B, Hauger O (2014). Standardization of selection criteria for percutaneous image-guided cryoablation of recurrent soft-tissue sarcomas. Diagn Interv Imaging.

[28] Cornelis F, Havez M, Lippa N, Al-Ammari S, Verdier D, Carteret T (2013). Radiologically guided percutaneous cryotherapy for soft tissue tumours: A promising treatment. Diagn Interv Imaging.

[29] Erinjeri JP, Clark TW (2010). Cryoablation: mechanism of action and devices. J Vasc Interv Radiol.

